# Supplementary material for: Phenotypic and Genotypic Characterization of 171 Patients with Syndromic Inherited Retinal Diseases Highlights the Importance of Genetic Testing for Accurate Clinical Diagnosis
Source: Genes (Basel). 2025 Jun 26;16(7):745. doi: 10.3390/genes16070745 (PMC12295353; doi:10.3390/genes16070745)
Supplement: Supplementary file 1 [file genes-16-00745-s001.zip › supplementary Figure S1.pdf]

Supplementary Figure S1.

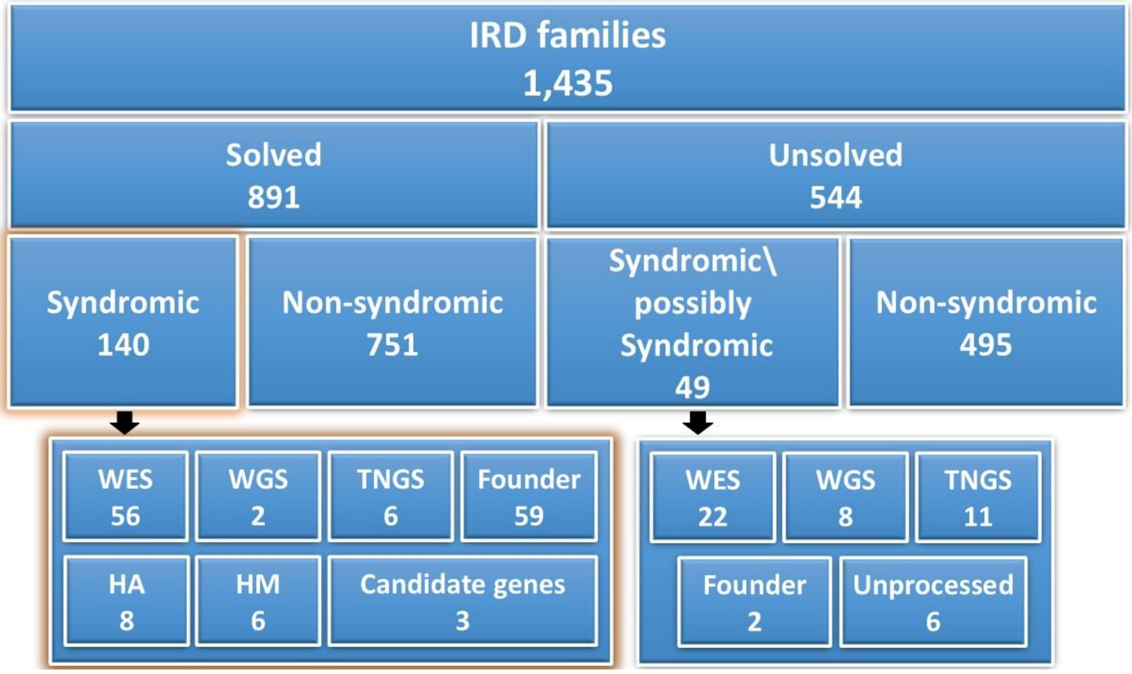

Composition of the entire IRD cohort and methods used for genetic analyses of syndromic IRD patients. HA, haplotype analysis; HM, homozygosity mapping; IRD, inherited retinal diseases; TNGS, targeted next-generation sequencing; WES, whole exome sequencing; WGS, whole genome sequencing.
