# Supplementary material for: Phenotypic and Genotypic Characterization of 171 Patients with Syndromic Inherited Retinal Diseases Highlights the Importance of Genetic Testing for Accurate Clinical Diagnosis
Source: Genes (Basel). 2025 Jun 26;16(7):745. doi: 10.3390/genes16070745 (PMC12295353; doi:10.3390/genes16070745)
Supplement: Supplementary file 1 [file genes-16-00745-s001.zip › Supplementary Figure S2.pdf]

Supplementary Figure S2.

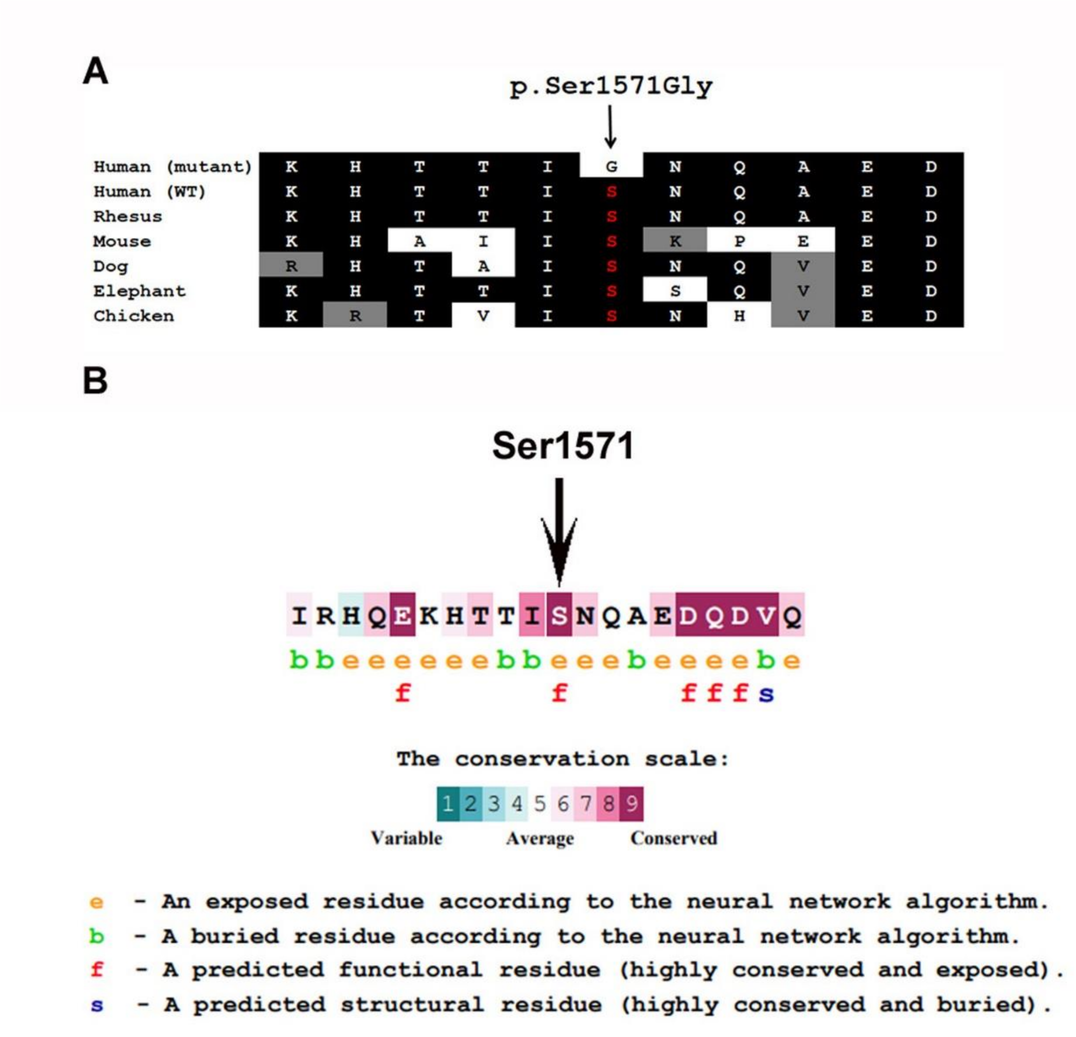

Evolutionary conservation of KATNIP serine 1571. **(A)** Multiple sequence alignment of the region spanning the Ser1571 amino acid of the KATNIP protein (highlighted in red and marked by an arrow) in various organisms. Conserved amino acids are indicated by a black background. Similar amino acids are indicated by a gray background. Also shown is the predicted sequence of the mutant protein harboring the p.Ser1571Gly variant (first line). **(B)** Analysis of KATNIP amino acid conservation across species. The analysis was performed with The ConSurf Server (https://consurf.tau.ac.il/ consurf_index.php) based on 150 KATNIP orthologues. e, exposed amino acid; b, buried amino acid; f, functionally important amino acid (conserved and exposed); s, structurally important amino acid (conserved and buried).
